# The Effect of Few-Layer Graphene on the Complex of Hardness, Strength, and Thermo Physical Properties of Polymer Composite Materials Produced by Digital Light Processing (DLP) 3D Printing

**DOI:** 10.3390/ma16031157

**Published:** 2023-01-29

**Authors:** Sergey Kidalov, Alexander Voznyakovskii, Aleksei Vozniakovskii, Sofia Titova, Yvgenii Auchynnikau

**Affiliations:** 1 Ioffe Institute, 194021 St. Petersburg, Russia; 2 Institute of Synthetic Rubber, 198035 St. Petersburg, Russia; 3 Department of Logistics and Management Methods, Yanka Kupala State University of Grodno, 230023 Grodno, Belarus

**Keywords:** photopolymer resin, few-layer graphene, FLG, DLP, 3D printing, hardness, bending strength, Charpy impact strength, thermal conductivity

## Abstract

The results of studying the effect of particles of few-layer graphene (FLG) synthesized by self-propagating high-temperature synthesis (SHS) on the complex of strength and thermo physical properties of polymer composite products obtained by digital light processing (DLP) 3D printing are presented. It was discovered to achieve an increase in thermophysical and strength parameters of polymers modified by FLG compared with samples made on the unmodified base resin. This result was achieved due to low defectiveness, namely the absence of Stone–Wales defects in the structure of FLG due to the homogeneous distribution of FLG over the volume of the polymer in the form of highly dispersed aggregates. It was possible to increase hardness by 120%, bending strength by 102%, Charpy impact strength by 205%, and thermal conductivity at 25 °C by 572% at concentrations of few-layer graphene of no more than 2 wt. %.

## 1. Introduction

Currently, there are many 3D printing methods, one of which is the digital light processing (DLP) method. This method is based on the layer-by-layer curing of photopolymer resin and is an evolution of the stereo lithography (SLA) method. Although the principle of the SLA method itself became known as early as 1986 [1], two years earlier than the fused deposition modeling (FDM) method [2], it was the FDM method that became the most popular 3D printing technique due to much cheaper 3D printers. DLP 3D printers became much cheaper, which made them available to almost everyone. However, although the DLP method makes it possible to obtain products with high accuracy, low roughness, and relatively quick, products made from photopolymer resins have relatively lower strength characteristics than products obtained by the FDM method.

One of the most promising ways to improve the properties of final products obtained by DLP 3D printing is the use of composite materials [3]. Metal nitrides [4], metal oxides [5], carbon black [6], lignin [7], their mixtures [8], etc., are used as fillers. By combining the properties of the matrix and the filler, researchers obtain final products with higher properties.

To improve the properties of final products, researchers also actively add various carbon nanomaterials, including graphene nanostructures, to the initial photopolymer resins [9]. Interest in graphene nanostructures is due to their record-breaking characteristics. It should be noted that the thermal conductivity of single-layer graphene is about 5000 W/(m × K) [10], and Young’s modulus is 1 TPa [11]. These characteristics allow you to get better results than when using classic fillers. These characteristics will enable you to get better results than when using traditional fillers.

For example, in [12], the authors use no more than 2 wt. % of graphene sheets and increased the tensile strength by 2.19 times compared to the original rubber. The authors also noted that the use of graphene nanosheets turned out to be more efficient than using standard filler—polyaniline fibers, with which only 1.41 times growth was achieved. In [13], the authors increased the flexural modulus and fracture toughness by 14% and 28% from neat resin, respectively, using 0.5 wt. % graphene nanoplates (GNP).

However, the use of graphene nanostructures does not always lead to the expected increase in the properties of the final composites. In [14], the authors showed that the introduction of 0.5 and 1 wt. % GNP only resulted in a deterioration in Young’s modulus, tensile strength, and other strength characteristics compared to pure rubber.

The reason for such different results in different groups of researchers can be explained by using graphene nanostructures with varying degrees of defectiveness. Defects significantly affect the final properties of the graphene nanostructures themselves [15] and, accordingly, the properties of composites modified with graphene nanostructures.

These defects include Stone–Wales defects. Stone–Wales defect is a crystallographic defect in carbon nanotubes, graphene, and other crystals with a hexagonal crystal lattice appearing when one of the C–C bonds is rotated through an angle of 90°; as a result of which, four hexagons of carbon atoms are converted into two heptagons and two pentagons [16]. In [17,18,19], the authors, using the method of molecular dynamics modeling, showed that Stone–Wales defects could significantly worsen the strength and thermophysical properties of graphene. In turn, the deterioration of graphene’s strength and thermophysical properties will worsen the final properties of polymer composites modified with graphene nanostructures [20]. However, there are no works in the literature where the effect of Stone–Wales defects in graphene nanostructures on their efficiency in polymer composites were experimentally evaluated.

In our previous work [21], we showed the possibility of synthesizing FLG under self-propagating high-temperature synthesis (SHS) conditions from cyclic biopolymers. An important advantage of FLG synthesized by this method is the absence of Stone–Wales defects [22], which is almost inevitable in synthesis by other methods. We found that FLG nanostructures synthesized by the SHS method could significantly improve nitrile butadiene rubber’s complex strength and thermos physical properties [23].

This work aimed to evaluate the effectiveness of graphene nanostructures that do not contain Stone–Wales defects when used as a modifying additive in creating products using DLP 3D printing compared to graphene nanostructures containing Stone–Wales defects.

## 2. Materials and Methods

### 2.1. Raw Materials

A commercial photopolymer resin of the Anycubic brand (405 nm, clear, China) was taken as the starting material for obtaining products using the DLP 3D printing method. According to the manufacturer, the resin consists of the following components: polyurethane acrylate (30–60%, CAS №51852-81-4, acrylate monomer (10–40%, CAS №29590-42-9, and photoinitiator (2–5%, CAS №106797-53-9).

The particles of FLG synthesized by the SHS method were taken as a modifying additive. The initial biopolymer (starch, analytical grade) was mixed with the oxidizing agent (ammonium nitrate, analytical grade) in a 1-to-1 ratio using a drunk barrel homogenizer for 15 min (60 rpm). Then, the resulting mixture was placed in a reactor and heated to a temperature of 220 °C (initialization of SHS synthesis). The procedure for obtaining FLG is described in detail in [21,24].

Graphene oxide (GO) synthesized by a modified Hammers method [25], which was treated with hydrazine to obtain reduced graphene oxide (rGO), was also taken as a modifying additive.

### 2.2. Stone–Wales Defects Concentration Measurement

To detect the concentration of Stone–Wales defects, we used our own technique based on the reaction of [4 + 2]-cycloaddition (Diels–Alder reaction). A mixture of α-methylstyrene and o-xylene taken in equal amounts was added to a suspension of carbon nanostructures in toluene to carry out the diene synthesis reaction. Control over the passage of the reaction was carried out by gas–liquid chromatography (GLC). Chromatographic studies were carried out using a Clarus 500 gas chromatograph. Research parameters: column temperature −145 °C; detector temperature −250 °C; evaporator temperature −250 °C; and gas rate −30 mL/min. A mixture of α-methylstyrene (basic reagent) with o-xylene (standard) was added to a suspension of rGO/FLG in toluene with vigorous stirring. The resulting suspension was placed on a magnetic stirrer. Samples of the mixture, taken every three hours, were injected into the chromatography column, and the ratios of α-methylstyrene/o-xylene in the mixture were determined. According to the ratio of peak areas of o-xylene/α-methylstyrene for each sample, it was concluded that the reaction was proceeding. The criterion for the reaction was a consistent decrease in the content of α-methylstyrene in the suspension. To eliminate the systematic error of the experiment, we specially set up a blank experiment (there was no α-methylstyrene in the solution), which showed the absence of sorption of o-xylene on the surface of the selected series of nanocarbons. The technique is described in detail in [22].

### 2.3. Specific Surface Area Measurement

Specific surface areas of synthesized samples were determined using multilayer ad-sorption on an ASAP 2020 analyzer (Norcross, GA, USA). Nitrogen was used as the adsorbate. The sample preparation was performed according to the standard procedure of heating the samples in a vacuum at 300 °C for three hours before the measurements. The measurement error did not exceed 3%.

### 2.4. SEM Studies

SEM studies of the morphology and structure of polymer composites samples (fractured surfaces) were carried out using the TESCAN Mira-3M (Brno, Czech Republic, 15 kV).

### 2.5. Measurement of Dispersion of FLG Particles

Particle dispersity was measured on a Zetasizer nano ZS instrument (dynamic light scattering method). To measure dispersion of FLG particles, suspensions of FLG in a photopolymer resin were taken, and diluted, if necessary, to a concentration of 0.25 wt. %.

### 2.6. Obtaining Products from Photopolymer Resins with the Addition of FLG

**Figure 1:**
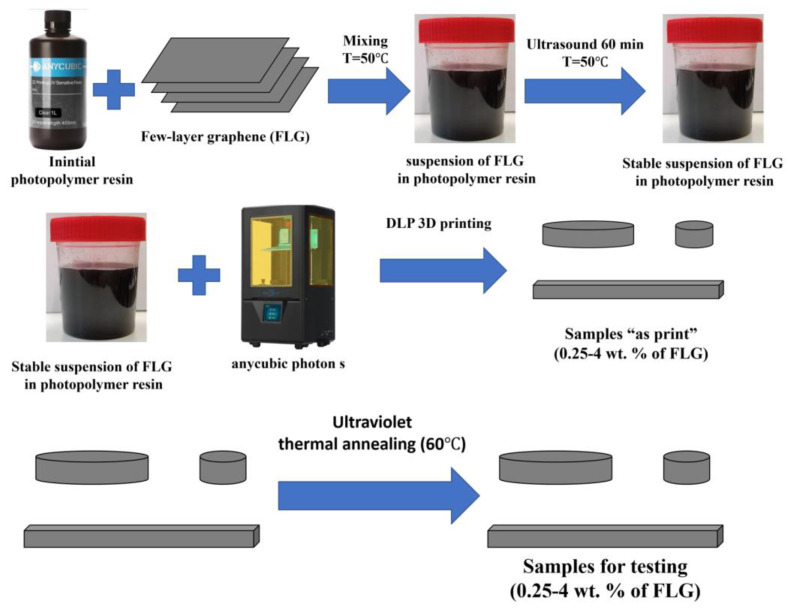
Scheme for obtaining samples from photopolymer resins with the addition of FLG.

The scheme for obtaining samples from photopolymer resins with the addition of FLG is shown in Figure 1.

In the initial photopolymer resin heated to 50 °C in uniform portions (0.1 of the entire sample), FLG powder was sequentially added with constant stirring using an overhead stirrer (500 rpm).

The concentration of the additive ranged from 0.25 to 4 wt. %, which corresponded to 0.475 to 7.6 vol. %.

**Figure 2:**
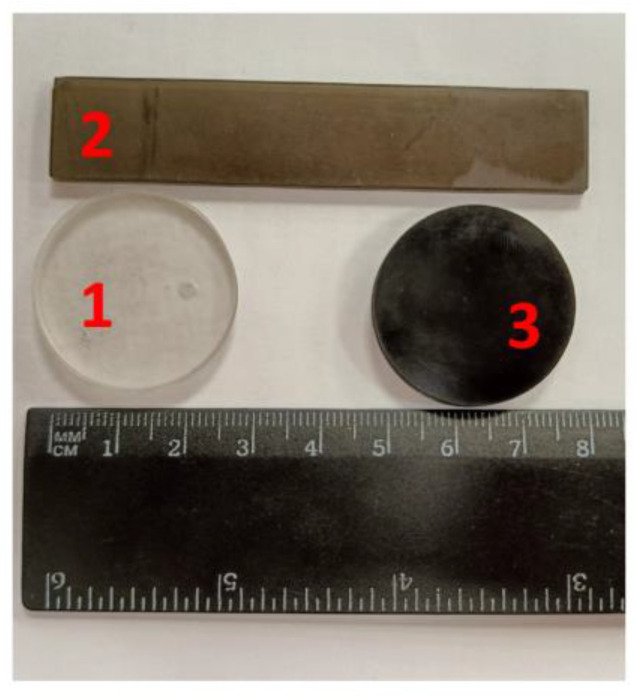
Samples of products obtained by DLP 3D printing: 1—initial resin, 2—0.25 wt. % FLG, and 3—2 wt. % FLG.

Then, the resulting suspension was kept in the ultrasound field for 1 h (ultrasonic tub, 22 kHz) while maintaining the temperature at 50 °C until a stable suspension was obtained. The cooled photopolymer resin with FLG was placed in the “Anycubic Photon S” DLP 3D printer, and samples of the required sizes were made. Printing parameters: illumination layer thickness of 50 microns and exposure time of 6 s. Figure 2 shows typical synthesized samples.

Then the obtained samples were sequentially subjected to UV treatment for 1–2 h and thermal annealing for 1–2 h (70 °C).

Samples of composites with rGO (2 mass. %) were fabricated similarly.

### 2.7. Viscosity Measurement

The dynamic viscosity of stable suspensions of FLG in photopolymer resins was measured on a rotational viscometer NDJ-9S (XZBELEC, Shenzhen, China); the shear rate in our case is 30 s^−1^, using a thermostat (WEST TUNE, Hangzhou, China), with a temperature maintenance accuracy of ±0.1 °C.

### 2.8. Hardness Test

The measurement of hardness by the Brinell method (ISO 506-81) was carried out on a hardness tester Metrotest ITB-3000 AM (steel ball with a diameter of 5 mm, load—62.5 kgf, and exposure time—120 s, Metrotest, Neftekamsk, Russia).

The dimensions of the prints were determined using an image analysis system. Based on this data, the hardness of the material was calculated.

Samples in the form of discs 30 mm in diameter and 5 mm thick were prepared for hardness testing.

### 2.9. Measurement of Flexural Strength

The flexural strength was measured on a PM-MG4 hydraulic press in accordance with ISO 178:2010. For flexural strength tests, samples were made in the form of beams with a length of 80 mm, a width of 15 mm, and a height of 3 mm. The flexural strength was measured under the dynamic loading of a flat specimen up to the point of failure. At the moment of failure, the load on the sample was recorded. The sample loading rate was 2 mm/min.

### 2.10. Measurement of Charpy Impact Strength

Data on the impact strength of composites (impact in the rib) were obtained on a Koper pendulum KMM-50 (RF) by ISO 179-1:2010. Charpy impact strength was measured by evaluating the absorbed energy of a hammer impact on a specimen on the holder. The test setup was a pendulum impact tester, which was installed at a certain height above the sample. The impact on the sample was made by means of a fall of a copra. The impact energy absorbed during the breaking of the sample was proportional to the difference in height from which the copra fell and the height to which it was able to rise by inertia after breaking the sample.

Samples were prepared in the form of beams (without notch) with a length of 80 mm, a width of 10 mm, and a height of 4 mm. The distance between the supports was 60 mm.

### 2.11. Measurement of Thermal Conductivity and Heat Capacity

Thermal conductivity and heat capacity were measured using a DXF-200 flash method (using a xenon lamp) at 25 °C. The samples were cylinders 10 mm in diameter and 1 mm thick. One side of the sample was covered with a thin layer of graphite paint (for the complete absorption of the flash energy) to improve the experiment’s accuracy. The heat capacity measurement accuracy was ±7%, and the thermal conductivity measurement accuracy was ±5%.

## 3. Results and Discussion

**Figure 3:**
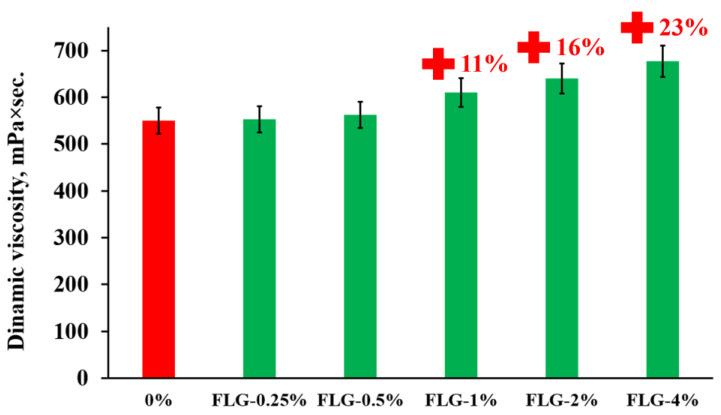
Dependence of the dynamic viscosity at 25 °C of stable suspensions of photopolymer resins after ultrasonic sonication at 50 °C depending on the concentration of FLG.

Dynamic viscosity is an important parameter that affects the usability of photopolymer resin. The results of measuring the dynamic viscosity of the resulting FLG suspensions in the photopolymer resin are shown in Figure 3.

**Figure 4:**
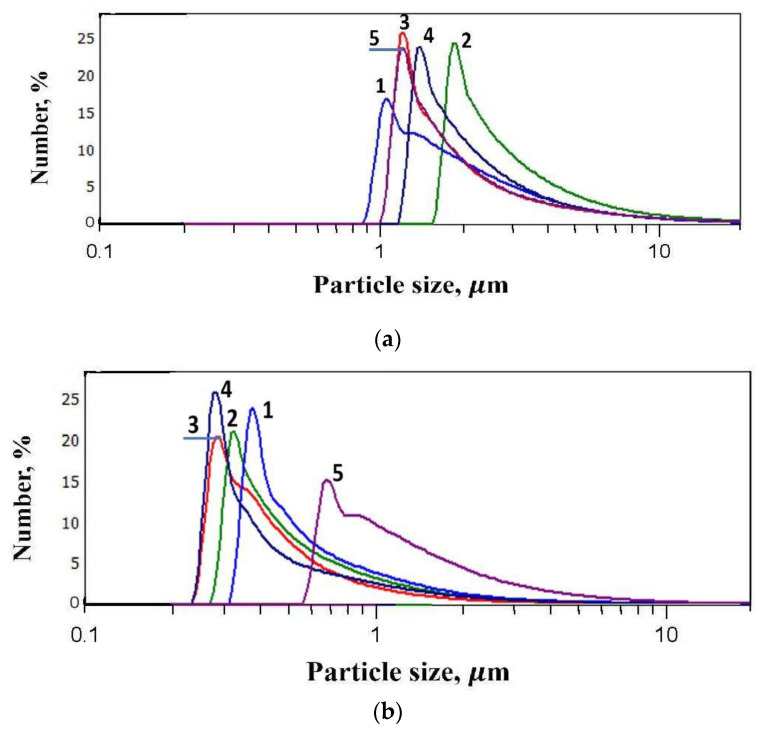
Results of measurements of the dispersion of FLG particles in a photopolymer resin. (**a**)—without ultrasonic treatment, 1—0.25 wt. %, 2—0.5 wt. %, 3—1 wt. %, 4—2 wt. %, and 5—4 wt. %; (**b**)—ultrasonic treatment at 50 °C, 1—0.25 wt. %, 2—0.5 wt. %, 3—1 wt. %, 4—2 wt. %, and 5—4 wt. %.

As can be seen from Figure 3, the introduction of FLG at a concentration of more than 1 wt. % leads to a slight increase in dynamic viscosity. The introduction of graphene nanostructures often leads to a greater increase in dynamic viscosity at such concentrations, as was shown in [26,27]. Therefore, a study was made of the dispersion of FLG particles in a photopolymer resin before and after ultrasonic sonication at 50 °C. The measurement results are shown in Figure 4.

As can be seen from Figure 4, the dispersion of FLG particles after ultrasonic treatment changes significantly from 1.2–1.4 µm to 250–300 nm. It should be noted that a similar measurement of dispersion occurs at other concentrations (0.25; 0.5; 2; and 4 wt. %).

Such a significant increase in dispersion, in combination with a substantial decrease in the viscosity of the photopolymer resin due to heating, makes it possible to evenly distribute FLG particles throughout the volume, which leads to a relatively low increase in dynamic viscosity.

Post-processing (ultraviolet irradiation and thermal annealing) of 3D printed samples can significantly improve the final complex of product properties. Therefore, we investigated the effect of a combination of post-processing techniques (UV treatment and annealing at 70 °C) and their duration on the hardness of the final products. The results are presented in Table 1.

As can be seen from Table 1, 1 h UV treatment followed by 1 h annealing gave the best results, so this post-processing was used for all further samples.

**Figure 5:**
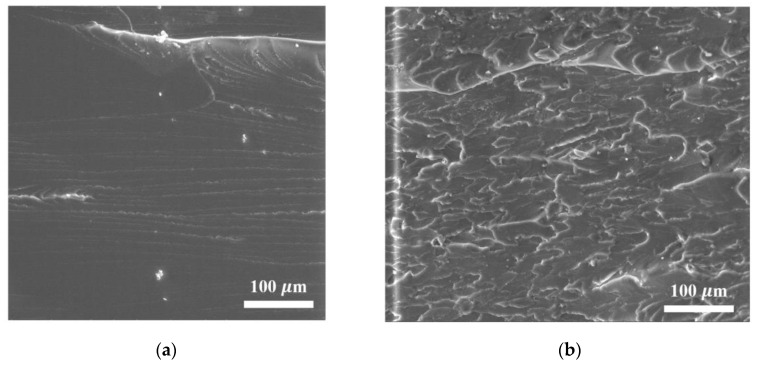

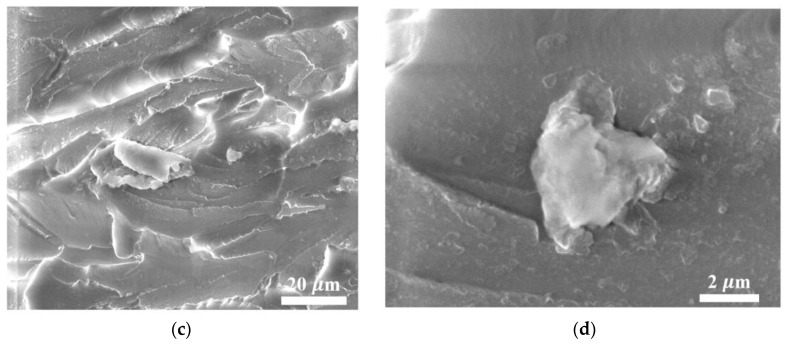
SEM images of (**a**) cleavage sample (fractured surfaces) from the original resin (linear scale 100 µm); (**b**) modified 1 wt. % FLG (linear scale 100 µm); (**c**) modified 1 wt. % FLG (linear scale 20 µm); and (**d**) modified 1 wt. % FLG (linear scale 2 µm).

Figure 5 shows the results of studying the structure of the obtained products by the SEM method (fractured surfaces).

As seen from Figure 5b–d, FLG particles in the polymer composite are distributed in the form of aggregates of various fineness, which is consistent with the result of measuring fineness by the DLS method.

**Figure 6:**
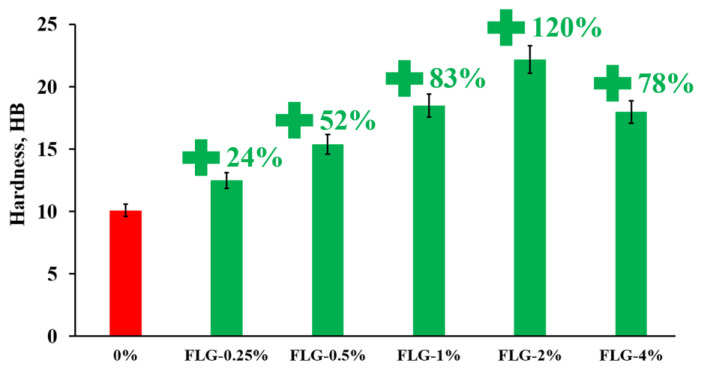
Brinell hardness of samples depends on the concentration of FLG.

Figure 6 shows the results of measuring the Brinell hardness of the synthesized samples.

As seen in Figure 6, the introduction of FLG makes it possible to increase the Brinell hardness up to 120% compared to a pure sample at an FLG concentration of 2 wt. %. A further increase in the concentration of FLG does not lead to a further increase in hardness.

**Figure 7:**
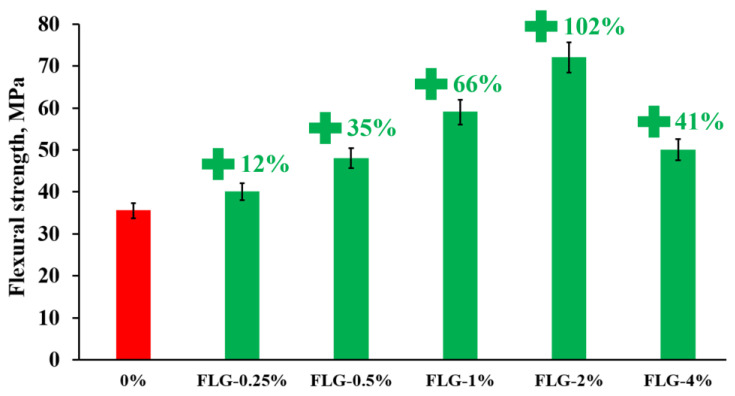
Flexural strength of samples depending on the concentration of FLG.

**Figure 8:**
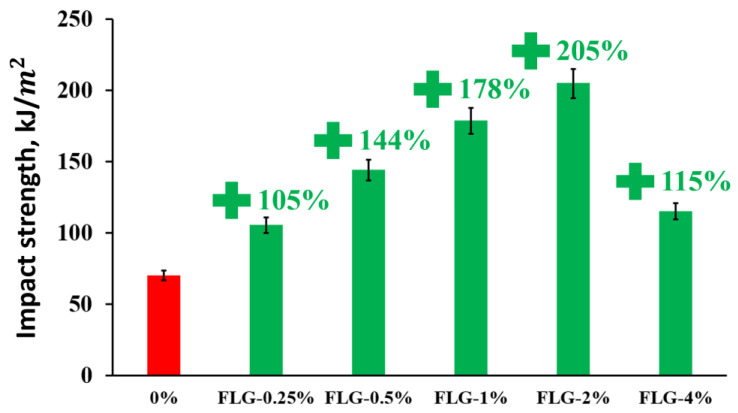
Charpy impact strength of the samples depending on the concentration of FLG.

Figure 7 and Figure 8 show the results of measuring the flexural strength of the obtained composites.

As seen in Figure 7, the introduction of FLG makes it possible to increase the flexural strength up to 102% compared to a pure sample at a FLG concentration of 2 wt. %. A further increase in the concentration of FLG does not lead to a further increase in the flexural strength.

As seen in Figure 8, the introduction of FLG makes it possible to increase the Charpy impact strength up to 205% compared to a pure sample at an FLG concentration of 2 wt. %. A further increase in the concentration of FLG does not lead to a further increase.

The data on the effect of FLG on the complex strength properties of final products (hardness, bending strength, and impact strength) show that FLG can significantly improve the properties of final composites.

**Figure 9:**
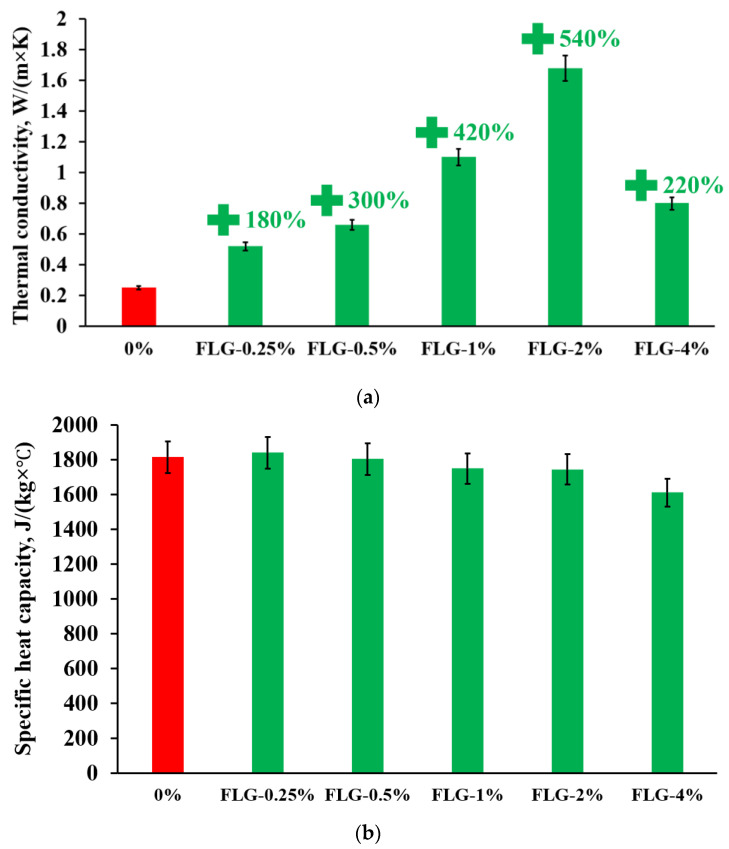
Dependence of thermal conductivity (**a**) and specific heat capacity (**b**) of synthesized samples on FLG concentration at 25 °C.

Figure 9 shows the results of measuring the synthesized products’ thermal conductivity and heat capacity.

As seen in Figure 9, the introduction of FLG makes it possible to increase the thermal conductivity up to 540% compared to a pure sample at an FLG concentration of 2 wt. %.

However, the synthesized samples’ specific heat capacity remains unchanged (within the method error). This is because the heat capacity of graphene nanostructures is estimated at 700 J/(kg × °C) [28], which leads to a gradual decrease in the heat capacity of the composite with an increase in the proportion of FLG.

The increase in the strength and thermophysical properties of the polymer matrix is primarily due to the high characteristics of the FLG itself, which makes it possible to lower the percolation threshold and obtain good results even at low FLG concentrations [29,30]. However, to realize its characteristics, it is necessary to evenly distribute the modifying additive over the volume of the polymer matrix, for which researchers use several techniques, such as mechanical mixing, ultrasonic sonication, etc. [31]. Figure 4 shows that when adding 4 wt. % FLG, the dispersity of its particles sharply decreases compared to other concentrations and indicates the aggregation of FLG particles, which directly affects the final properties of polymer composites. In a review [32], the authors noted that the fineness of particles significantly affects the final strength and thermal properties of polymer composites.

To compare the obtained experimental data with various models of thermal conductivity of composite materials, the volume fractions of FLG in the synthesized composite were calculated from the measured density of composites and the mass fraction of FLG.

For the simplest assessment of the thermal conductivity of composite material, a geometric model can be used.

The volume fractions of FLG in the synthesized composite were calculated from the measured density of composites and the mass fraction of FLG to compare the obtained experimental data with various models of thermal conductivity of composite materials.

A geometric model can be used for the simplest assessment of the thermal conductivity of composite material [33].

(1) $$\lambda_{c}=\lambda_{m}{}^{v_{m}}\times \lambda_{f}{}^{v_{f}}$$

where λ_c_—thermal conductivity of composite material (W/(m K)), λ_m_ = 0.25—thermal conductivity of the matrix (W/(m K)), λ_f_—thermal conductivity of the filler (W/(m K)), *v*_m_—volume fraction of the matrix, %, and *v*_f_—volume fraction of the filler, %.

To calculate the theoretical values of the thermal conductivity of polymer composites, researchers often use the Maxwell model [34]:

λ_c_ = λ_m_ × (λ_f_ + 2 × λ_m_ − 2 × *v*_f_ × (λ_m_ − λ_f_))/(λ_f_ + 2 × λ_m_ + *v*_f_ × (λ_m_ − λ_f_)). (2)

However, the Maxwell model assumes that the filler particles are spherical. Therefore, to account for the thin sheets shape of the filler FLG particles, we use the Maxwell–Burger–Eiken (MBE) model [35]:

λ_c_ = (λ_m_ × (1 − (1 − λ_f_/λ_m_) × L × *v*_f_)/(1 + (L − 1) × *v*_m_) (3)

where L = (λ_m_ + 2 × λ_f_)/(3 × λ_m_) is the coefficient, taking into account the thin sheets shape of the FLG filler particles.

It should be noted that although the thermal conductivity of graphene is estimated at up to 5000 W/(m × K), aggregates of graphene particles have orders of magnitude lower thermal conductivity, which is due to energy losses at particle boundaries. This fact is one of the reasons for the discrepancy between theoretical calculations and experimental data [36]. Using experimental data on the thermal conductivity of the composite with 1 wt. % FLG and the models described above, the thermal conductivity of aggregates of FLG particles was evaluated (Table 2).

**Figure 10:**
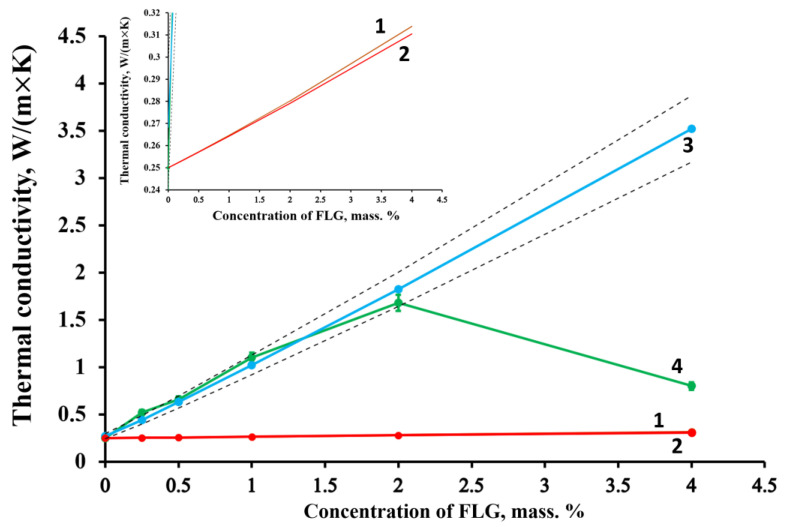
Comparison of experimental data with the results of calculations by models: 1—geometric model; 2—Maxwell model; 3—MBE model; and 4—experimental data. The dotted line shows a 10% deviation from the MBE model.

As seen from Table 2, the change in the thermal conductivity of the filler for the geometric model and the Maxwell model practically does not change the calculated result. In the case of the MBE model, the thermal conductivity value drastically changes the final calculated value of the thermal conductivity of the composite. Having carried out an additional selection of values for the MBE model, it was found that the thermal conductivity of FLG particle aggregates can be estimated at 40 W/(m × K), which was taken for further calculations. The calculation results are shown in Figure 10.

As can be seen from Figure 10, in contrast to the geometric model and the Maxwell model, which give greatly underestimated values compared to the experimental data, the MBE model gives an acceptable agreement with the experimental data up to 2 wt. % of FLG. These results are because, in the geometric model and the Maxwell model, it is assumed that the filler particles have a spherical shape and do not interact with each other. In contrast, the MBE model is where the lamellar shape of the filler particles is taken into account.

Comparing the experimental data with the calculated values, it can be seen that while the FLG are in a highly dispersed state, the experimental results coincide with model calculations using the MBE model and the thermal conductivity of the composite as a whole is determined by the mass fraction of FLGs, as well as their thermophysical properties. A sharp deterioration in thermal conductivity occurs with a decrease in the dispersion of FLG particles due to their aggregation (Figure 4).

To reveal the effect of Stone–Wales defects in graphene nanostructures on the final properties of polymer composites, we measured the specific surface area and the concentration of Stone–Wales defects for a sample of FLG and rGO (Table 3).

**Figure 11:**
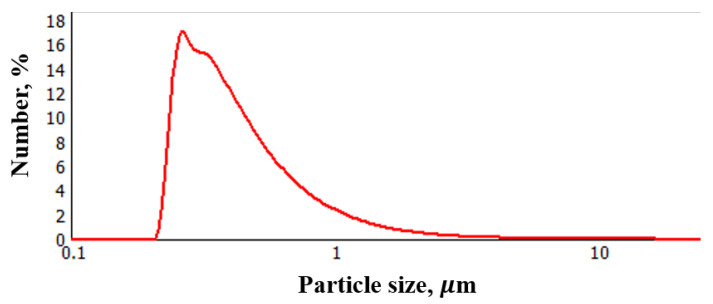
Results of measurements of the dispersion of rGO (two mass. %) particles in a photopolymer resin.

As can be seen from Table 3, the samples have a comparable specific surface area; however, in contrast to FLG, SE defects were found in the rGO samples. The dispersion of rGO particles is also equal to FLG particles and amounts to 240–320 nm (Figure 11).

**Figure 12:**
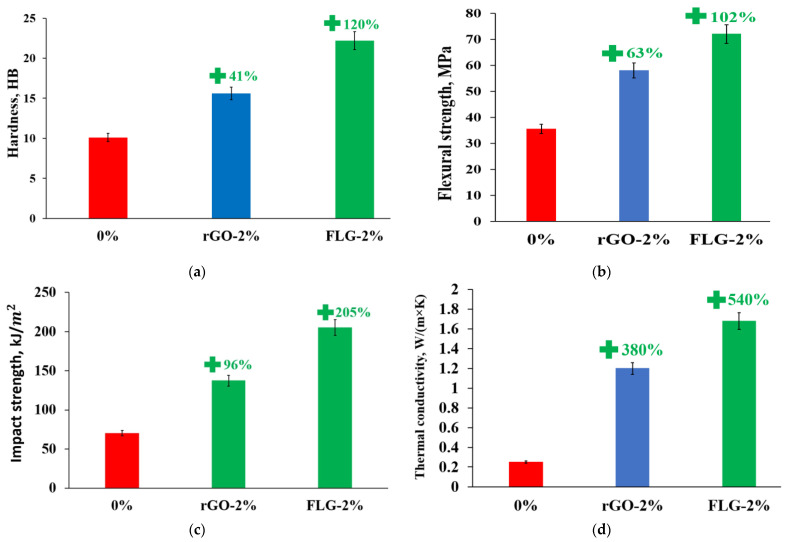
Comparison of the effectiveness of FLG and rGO: hardness (**a**), flexural strength (**b**), Charpy impact strength (**c**) and thermal conductivity (**d**).

Figure 12 compares the efficiency of samples of polymer composites modified with rGO/FLG and obtained under the same conditions.

As seen in Figure 12, FLG changes the properties of the polymer matrix much more efficiently than rGO at the same concentrations. It should be noted that the methods of sample preparation and synthesis of polymer composites were the same, and the modifying additives have similar characteristics (specific surface and dispersion). Based on this, it can be assumed that the high efficiency of FLG is due to the absence of Stone–Wales defects, which coincides with the simulation results [17,18,19,20].

## 4. Conclusions

FLG particles synthesized under the conditions of the SHS process showed themselves to be effective fillers capable of significantly increasing the complexity of strength and thermophysical properties of polymer composite products obtained by the DLP 3D printing method. As a result, it was possible to increase hardness and flexural strength by two times, impact strength by three times, and thermal conductivity at 25 °C by six times at concentrations of FLG 2 wt. %.

For the first time, it was experimentally confirmed that Stone–Wales defects in graphene nanostructures significantly negatively affect the strength and thermophysical properties of polymer composites.

Since the final properties of polymer composites strongly depend on the defectiveness of graphene nanostructures, studies are needed to experimentally evaluate the effect of other types of defects on the properties of polymer composites.

## Figures and Tables

**Table 1:** Dependence of Brinell hardness on post-processing techniques and their duration.

Post-Processing Technique	Brinell Hardness	Change, %
No processing	5.6 ± 0.3	0
1 h UV	7.4 ± 0.4	32
1 h annealing	6.1 ± 0.3	9
1 h UV + 1 h annealing	10.6 ± 0.5	89
1 h UV + 2 h annealing	8.8 ± 0.4	57
2 h UV + 1 h annealing	10.2 ± 0.5	82
2 h UV + 2 h annealing	8.2 ± 0.4	46

**Table 2:** The results of calculations of the thermal conductivity of the composite with 1 wt. % FLG for various models depending on the thermal conductivity of FLG aggregates.

*v*_m_, %	*v*_f_, %	λ_m_,	λ_f_,	λ_c_, Geometric Model	λ_c_, Maxwell Model	λ_c_, MBE Model	λ_c_, Experimental Value
%	%	W/(m × K)					
0.981	0.019	0.25	0.5	0.265	0.254	0.27	1.1 ± 0.05
0.981	0.019	0.25	5	0.265	0.263	0.41	
0.981	0.019	0.25	50	0.265	0.264	0.86	
0.981	0.019	0.25	500	0.265	0.265	1.86	
0.981	0.019	0.25	5000	0.265	0.265	10.12	

**Table 3:** Surface area and Stone–Wales defects concentration in FLG and rGO samples.

Sample	Surface Area, m^2^/g	Stone–Wales Defects Concentration (mol/m^2^)
FLG	660	0
rGO	580	3.6 × 10^−5^

## Data Availability

The data presented in this study are openly available in [21,22].
